# How digital health documentation transforms professional practices in primary healthcare in Denmark: A WPR document analysis

**DOI:** 10.1111/nin.12499

**Published:** 2022-05-10

**Authors:** Julie Duval Jensen, Loni Ledderer, Kirsten Beedholm

**Affiliations:** ^1^ Department of Public Health Aarhus University Aarhus C Denmark

**Keywords:** digital, document analysis, documentation, EHR, practice, primary healthcare, problem representation, WPR

## Abstract

Historically, recordkeeping has been an essential task for health professionals. Today, this mandatory task increasingly takes place as digital documentation. This study critically examines problem constructions in practical documents on digital documentation strategies in Danish municipal healthcare and how these problem constructions imply particular solutions. A document analysis based on the approach presented in Bacchi's “What's the problem represented to be?” was applied. Forty practical documents in the form of guidelines, strategies, and quality control documents were included. The analysis uncovered three problem representations: lack of coherence between health services in a complex healthcare system, lack of assessable data for management and political prioritization, and inefficiency in the healthcare system. The proposed solution is a digitalized and standardized practice that transforms recordkeeping in the municipalities. However, municipal healthcare is at risk of being fragmented due to digital documentation's focus on the organizational management of health with task‐oriented practices supplied by an anonymous health professional. We find that digital documentation functions as an organizational micromanagement approach that assigns the health professional a subject position as an employee acting according to the organization's framework rather than the profession's normative framework.

## 1 INTRODUCTION

This article presents part of a study whose overall aim was to explore digital documentation in primary healthcare in Denmark.

The analysis was conducted on practical documents (Bacchi & Goodwin, 2016; Prior, 2003) that were written with the purpose of offering rules, opinions, and advice on how to behave. The study was based on the assumption that practical documents construct recommended healthcare and affect health professionals' digital documentation practices.

## 2 BACKGROUND

Documentation has a long tradition in health professionals' practice. The first medical records date back to Egypt in 1600 BC (Doyle‐Lindrud, 2015; Evans, 2016). In the 1970s, a transition began as computer technology was introduced (Evans, 2016). By the end of the 1990s, almost all healthcare organizations in developed countries had a digital system that supported healthcare professionals (Watson, 2016). A key aspect of this digitalization is the Electronic Health Record (EHR) which is a vital component of health informatics (Braunstein, 2015). According to Petrakaki et al. (2016), healthcare documentation often relates to accomplished clinical procedures and informs health professionals about treatment and care. Today, almost all healthcare professionals worldwide document and communicate in the EHR (Menvielle et al., 2017). Historically, the term “recordkeeping” has been used in healthcare to define the process of submitting written information between health professionals. Over time, the term has been replaced by “documentation.” As we show in this article, this reflects a transformation of the meaning of documentation practices. For simplicity, the term “documentation” is used to designate both processes in this article.

### 2.1 Documentation in healthcare practice

Research indicates that there are many benefits for care, treatment, and patient experience when health professionals record their actions in journals. Studies show that healthcare documentation plays a vital role in patient safety and risk prevention (Blair & Smith, 2012; King et al., 2014), mainly when documentation in EHRs supports organizational workflow and provides health professionals with necessary knowledge (Eklöf et al., 2014). Even so, documentation procedures are not straightforward; a recent study showed that from 2016 to 2019, 69% of inspected institutions in Danish home care and care homes had insufficient documentation (Hertzum, 2021).

Other studies have focused on barriers to documentation and potential consequences. For example, Alexander (2015) examined how nursing assistants communicate using notes and pictures in addition to electronic records to prevent skin ulcers, while Stevenson (2018) concluded that unsuitable structures in EHRs resulted in workarounds when health professionals tried to ensure patient safety.

Research has also focused on topics related to the transformation from analog to digital documentation, for example, time‐saving issues (Baumann et al., 2018; McCarthy et al., 2019), the correlation between standardized frameworks and quality (Blair & Smith, 2012), digital documentation as a cost‐saving practice (Adler‐Milstein et al., 2013; Zlabek et al., 2011), and the relation between digital standardization and concrete practice (Johnson et al., 2012; Winman et al., 2012).

### 2.2 Digital documentation in Danish healthcare

Today, the digitalization of documentation is considered to be increasingly important worldwide (WHO, 2021). Denmark has one of the most digitized healthcare services globally (Sundheds‐og Ældreministeriet, 2018). The digital infrastructure in Denmark is the result of several digitalization strategies published by political actors at local and national levels (Den Digitale Taskforce, 2002; Kommunernes Landsforening, 2016; Regeringen, 2004; Regeringen, Danske Regioner, & Kommunernes Landsforening, 2013a; Regeringen, Danske Regioner, & Kommunernes Landsforening, 2013b; Regeringen et al., 2007; Sammenhængende Digital Sundhed i Danmark, 2008; Sundheds‐ og Ældreministeriet, 2018). The Danish public sector is almost entirely digital, with a digital infrastructure that extends across sectors and organizations (European Commission, 2020). Thus, all healthcare documentation occurs in EHRs in hospitals, general practices, and municipal healthcare. Health professionals use EHRs to share information and coordinate care and treatment across sectors and governmental areas.

Danish health services and home care are primarily tax‐funded. Services are free and delivered by different professional groups. The 98 Danish municipalities are responsible for primary health services and elderly care (Sundheds‐ og Ældreministeriet, 2017). Danish municipalities embarked on implementing a common method and standard for digital documentation called The Common Language Platform (CLP). KL‐Local Government Denmark, a political umbrella organization (Kommunernes Landsforening, 2021), has coordinated this new documentation method in all municipalities, creating common standards for compatibility. Thus, all suppliers of municipal healthcare systems have structured the EHR by the CLP. The implementation process was initiated by several practical documents in the form of strategies and instructions.

### 2.3 Focus and aim

In practice, management, and research, the most prevalent assumption seems to be that these practical documents represent “neutral” descriptions of pre‐existing problems that digitalization can help solve. In taking our epistemological starting point in the tradition of poststructuralism, we assume that digitalization creates the framework by which problems related to documentation are mentally and linguistically formed and perceived. A poststructuralist approach facilitates an analysis that is open to a plurality of practices, discourses, and possibilities. With this emphasis on contingencies, the subject of healthcare documentation can be perceived as an ongoing process. Thus, we expected that the practical documents take part in constructing the problems that digitalization is expected to solve, where problem representations construct digital documentation as a particular kind of practice. Against this backdrop, the aim of this article was to explore how problems are constructed in practical digital health documentation in the municipal health service and how these problems present particular solutions for health professionals.

## 3 METHODS

The study was conducted as a document analysis using Bacchi and Goodwin's "What's the problem represented to be?” (WPR) approach. According to this, practical documents are manufactured, consumed, and function in social practice to inform people about how to act (Bacchi & Goodwin, 2016, p. 34; Prior, 2003). The study was based on the poststructuralist premise that the solutions presented in practical documents are not just reactions to existing problems, but that the documents in presenting solutions construct problems as particular problems (Bacchi, 2009).

### 3.1 Documents

To identify relevant practical documents, a search was first performed on Danish web pages related to municipal digital documentation, for example, the Ministry of Health, KL‐Local Government Denmark, and the Digitalization Authority. Second, a snowball strategy was used that employed references from the initial search. This led to the selection of 40 documents published between 2000 and 2021 in the form of rules, guidelines, and norms of digital documentation in the Danish municipalities (see Supporting Information). The 40 documents were read through to gain an overview and contextual understanding of how digital documentation has evolved and been discussed over time. Based on this initial reading, four documents were selected for the primary analysis. The four documents were published in 2018 and 2019 and include the overall national digital health strategy (D1), guidelines for implementation of the strategy in municipalities (D2), and quality indicators for governmental control (D3–D4).

The four documents are:

1. D1: Digital Health Strategy 2018–2022 by the Danish Ministry of Health (2018).
  This provides an overall political strategy regarding the digitalization of healthcare and connects political priorities and funding. The strategy has multiple authors, with the Ministry of Health being the primary author. The Ministry of Health advises politicians and government, and frames proposals for legislation and strategy. This document was selected because it represents the overall vision, framing practice through negotiations, legislation, and so forth.
2. D2: The *Common Language Platform Method Handbook* by KL‐Local Government Denmark.
  The Method Handbook represents the concrete operationalization of legislation, policy, and strategy. It was developed by KL‐Local Government Denmark, an interest and member organization for Danish municipalities that reflects the common interests of the municipalities. KL‐Local Government Denmark negotiates a financial framework with the Danish parliament for the municipalities every year. The document provides a guideline for the CLP and is used in all municipalities. As such, the document is produced to guide practice and set the EHR's structure.
3. D3: Quality Control Indicators for Home Care Services and Nursing Homes 2019–2020 by the Danish Patient Safety Authority [Styrelsen for Patientsikkerhed] (2019a).
4. D4: Quality Control Indicators for Nursing Services 2019–2020 by the Danish Patient Safety Authority [Styrelsen for Patientsikkerhed] (2019b).

These two documents describe indicators for several factors used by Danish patient safety inspectors to assess quality in the organization and EHRs. The Danish Patient Safety Authority performs supervision and quality inspections in health institutions mainly based on legislation. The inspectors base the assessment on interviews, observations, and written text in the EHR.

These four documents (D1–4) are prescriptive and guide how the actors should behave. Such documents are regarded as key material and suitable for a WPR analysis (Bacchi and Goodwin, 2016, p. 18). The four documents are contemporary; especially, the method handbook and quality control indicators are currently used in Danish municipalities. As such, the four documents are examples of current governing policy texts that serve as a starting point to identify how problems are talked about and how the constructions of these problems lead to particular solutions in digital documentation practice. The remaining 36 documents are associated texts that increased our familiarity with the topic, provided contextual understanding, and acted as levers for reflection (Bacchi & Goodwin, 2016, p. 24).

### 3.2 The WPR—approach

**Figure 1:**
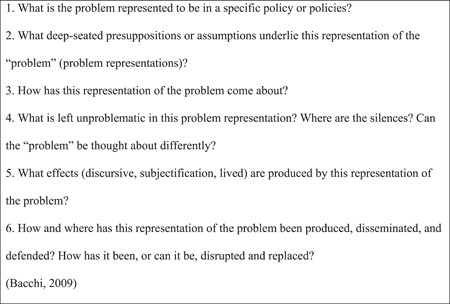
WPR—questions

The WPR approach (Bacchi, 2009; Bacchi & Goodwin, 2016) consists of six interrelated questions (Figure 1) that were applied repeatedly because the problem representations were “nested” (Bacchi, 2009, p. 21) into each another.

The first question helped us work backwards from the proposed digital solutions and strategies presented in the documents to examine which problems the solutions were expected to address. In line with Bacchi (2009), we did not question the proposed solutions but scrutinized the problematizations to reveal underlying understandings. The second question probes the underlying understandings more deeply. In this phase, the documents were examined for taken‐for‐granted assumptions underpinning the problem representations to reveal conceptual logics within the texts. The documents were searched for binaries, categories, and open‐ended objects. The third question is aimed at conducting a Foucault‐inspired genealogy (Bacchi, 2009, p. 10). This phase included all 40 documents. The analysis focused on how specific points and events affected problem representations over time and across different authors. The fourth question directed our analytical attention towards tensions or contradictions when “silences” showed us the limits of problem representations (Bacchi, 2009, p. 12; Bacchi & Goodwin, 2016, p. 22). A “silence” can suggest other conceptualizations of the problems and is identified by turning analytical attention to what is not written in the text or left unproblematic. As suggested by Bacchi (2009, p. 13), we went back to the second question to revisit the binaries used to simplify digitalization. The fifth question was examined by considering the effects of the identified problem representations. Three effects were analyzed: discursive effects, which limit what can be thought and said; subjectification effects, which constitute subjects in the discourse; and lived effects, which are material crystallizations of problem representations. With the sixth question, we investigated how problem representations achieved legitimacy and dominance among the problem representations.

**Figure 2:**
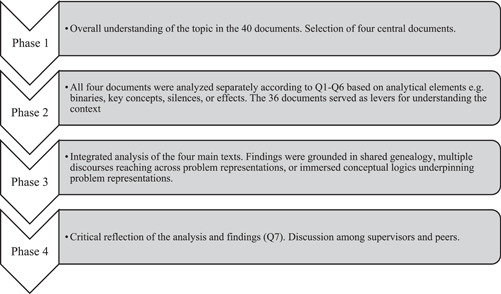
Schematic representation of the analysis process.

As a seventh step, Bacchi adds reflexivity on own embeddedness in the subject under investigation, which we addressed through ongoing reflections on our embeddedness in the conceptual logics of healthcare and documentation practice. A schematic representation of the phases of the analysis process can be seen in Figure 2.

## 4 ANALYSIS/FINDINGS

The analysis revealed three distinct but intertwined problem representations. The following section presents the problem representations (Q1), the underlying assumptions (Q2), genealogical features (Q3), and other possible representations/silences (Q4). The second section presents the effects of the problem representations and their impact (Q5) on healthcare documentation practice. Finally, the Discussion section addresses power relations (Q6) and further reflections.

### 4.1 The solution to lack of coherence, data, and inefficiency

In the four selected documents, digital healthcare documentation appears to be the solution to most problems in the healthcare system. However, three problems stand out distinctly:

(1) Lack of coherence between health services
(2) Lack of assessable data for management and political prioritization
(3) An inefficient healthcare system

#### 4.1.1 Lack of coherence between health services

Lack of coherence between health services in the healthcare system is represented as a problem in two ways (Sundheds‐ og Ældreministeriet, 2018, p. 8): as an issue related to citizens' individual experiences and as an organizational issue for the healthcare system. Coherence between health services appears to indicate quality in the healthcare system and is expected to be achieved by digital infrastructure, creating a digital network that contains digital highways for data and communication (Sundheds‐ og Ældreministeriet, 2018, p. 14). The suggested solution for both issues is digitalized “data” storage, which is expected to enable efficient information sharing across time and place: “[…] *we need to provide healthcare employees with easy, secure access to relevant knowledge so that they interact with the patient in the most expedient manner possible.”* 1 (D1, p. 18). It is assumed that coherence is achieved when knowledge and information regarding citizens are digitally shared among health professionals (Kommunernes Landsforening, 2002). Documentation is constructed as a professional tool that underpins professional practice and ultimately creates coherence in treatment and care across different professional actors and different times. For example, the Danish Patient Safety Authority states that “*help, care, and nursing [are] based on the professional description in the documentation”* (D4, p. 30).

This construction of the individual's experience of continuity as a direct correlation to health professionals' information sharing can be traced back to the first digitalization strategies. Thus, the tradition for recordkeeping has been repeated in legislations and guidelines regarding authorization (Indenrigs‐ og Sundhedsministeriet, 2005b; Sundheds‐ og Ældreministeriet, 2013) and quality improvement, as well as patient experiences (Den Digitale Taskforce, 2002; DSI, 2010; Sundheds‐ og Ældreministeriet, 2016). Common to the documents is an emphasis on the idea that written information leads to knowledge, subsequently leading to coherence, diverting attention from the physical presence of health professionals or other forms of collaboration as a source of information sharing. As such, words, phrases, or actions that relate to “meetings,” “team meetings,” “feedback,” or “handover” are not present in any of the central documents.

#### 4.1.2 Lack of assessable data for management and political prioritization

The second problem is lack of assessable data for management and political prioritization (D1, D2, D3, D4). The proposed solution is to standardize information in the EHR, as this is expected to enable data extraction. The assumption is that organizations need “*valid data*” (D2) to control costs and resources.

Generally, “data” are a relatively open concept that can be “*filled with different meanings*” (Bacchi, 2009, p. 8). In these documents, *“data*” are exclusively used for standardized and classified information and connotes instrumental and quantified healthcare. Via standardization, healthcare documentation is expected to transform into extractable data related to quality, economics, and statistics (D2). The assumption is that health professional documentation can be used to prove that certain healthcare activities have taken place, but only if it is standardized through “*classifications*” (D2, p. 19).

This problem representation can be traced back to the first digitalization strategy (Sundhedsministeriet, 1999) and the first CLP document (Kommunernes Landsforening, 2002), and has been stable in subsequent practical documents. Within the CLP, functional capacity is linked to numeric values, for example, the professional description: “*Eats when food is served and cut into pieces. Must be encouraged to eat and drink*” (Kommunernes Landsforening, 2002, p. 28), which is equivalent to functional capacity level 3. The numerical values were expected to enable "*credible data*” (Kommunernes Landsforening, 2002, p. 15) for politicians and administrators when negotiating the financial framework (Kommunernes Landsforening, 2002, p. 18). The financial agreement from 2005 between the government and the municipalities claims that financial difficulties can be solved by “*better documentation*” of the tasks and “*systematic evaluation*” of finances and efficiency (Finansministeriet, 2004, p. 16). What is referred to in this document is not recordkeeping but documentation as “proof of performance of tasks.” In the following year, 2006, the focus was on professional documentation in the form of knowledge about "*task solution*,” “*benchmarking*,” and “*achieving goals*” (Finansministeriet, 2006, pp. 10, 25). The articulation of the need for data accelerated with the so‐called partnership project between KL‐Local Government Denmark and 45 Danish municipalities in 2010. KL‐Local Government Denmark argued that data are necessary to achieve the needed funding for municipal healthcare:*“*[. .] *it has not been possible to obtain financial compensation for the municipalities, as the available documentation for the task increase has not been sufficient*” (Kommunernes Landsforening, 2010b, p. 2).

The assumption is that with the introduction of digital documentation methods, valid data will be obtained. There is a silencing of the idea that health professionals can simply opt out of following the structures, documenting instead in other classifications—or not documenting at all. The standardization of documentation is presented as the desirable scenario, disregarding the possibility that health professionals may choose not to follow this path.

#### 4.1.3 An inefficient healthcare system

The third problem representation is what we term "an inefficient healthcare system." The proposed solution is a standardized workflow in assessing, delivering, and evaluating care. The CLP document considers standardized workflow an essential element in the documentation method (D2). One of the defined goals of the CLP is “[. .]*increased efficiency and profitability*” (D2, p. 10).

Across the documents, the concept of efficiency is used differently. For example, in the Digitalization Strategy, inefficiency appears to be a problem for both health professionals and citizens: “*Digital health is thus about making everyday life for patients, relatives and employees work more efficiently, and making better use of the health service's resources”* (D1, p. 10). KL‐Local Government Denmark operationalizes this vision of digital health with the CLP document. First, citizens must be assessed for what is “*relevant*” (D2). It is not explicitly described for whom it may be relevant. Still, the criterion of “relevance” seems implicitly to be for the organizations, and services must be relevant for the citizens' recovery. Services have a specific aim in the form of an "*expected condition*” (D2) and are monitored continuously to assess whether services should continue or end. The CLP functions as an organizational algorithm that standardizes the care trajectory when citizens' needs are assessed via “*conditions*." Visitation of services takes its starting point from the service catalog, and the trajectory is planned with an “*expected condition*”(D2). The “service catalog” (D2, pp. 10, 37, 43–44) includes categories whose services the citizen can be assessed for receiving. Services from the individual municipality are framed with defined times that depend on the particular municipality's service level. Thus, organizational processes are expected to be managed and optimized by implementing a standardized and rigorous documentation method. The care trajectory is constructed and described as an organizational “*case*” (D2, pp. 43–44), almost detached from the citizen.

The problem representation of efficiency can be traced back to the first CLP document (Kommunernes Landsforening, 2002). Efficiency has been increasingly in focus over the years, especially with the political reform of the healthcare system in 2007 (Indenrigs‐ og Sundhedsministeriet, 2005a), resulting in a distribution of responsibility and increasing task loads in municipalities without financial compensation (Finansministeriet, 2004, 2006, 2014). In 2010, KL‐Local Government Denmark wrote: “*The municipalities face three significant challenges at the same time: fewer resources, more difficult recruitment, and expectations of higher quality in welfare services. The municipalities must find an answer to this, and one of the essential tools in the coming years will be digitalization*” (Kommunernes Landsforening, 2010a, p. 10).

It is silenced that documentation can occur in other media than digital, for example, on written paper notes in homes or offices. On the contrary, it appears that documentation has to be digital to be converted into useful data. With this, the documents construct a dichotomy between “digital practice,” which is explicitly described with positive connotations, and “analog practice,” which implicitly appears to be its negative counter‐image: “*The Common Language Platform is built around structuring and classifying data, which makes it easier to find the right place to document. When conditions and efforts are classified, it supports more uniform communication as well as the possibility of exchanging data within the municipality, between municipalities and between the municipality and other actors*” (D2, p. 10). The documents do not take account of other forms of communication or actions than the digital. Organizational activities, such as interdisciplinary team meetings or collaboration with families, are not described in any of the central documents.

### 4.2 Effects

The effects of these three problem representations are intertwined across the representations. Below, we present them in three main categories.

#### 4.2.1 A citizen's journey in a seamless system

One effect of this way of discursifying digital documentation is that the citizen's care trajectory is constructed as a journey in a supposedly seamless healthcare system. Information is constructed as a critical aspect for creating coherence, and is provided by the EHR. Health professionals are to create this coherence by always having knowledge of the citizens' previous and future contacts with the healthcare system, regardless of organizational boundaries. Both the Ministry of Health and the Danish Patient Safety Authority represent digital documentation as a professional tool that ensures patient safety and coherence. According to the Ministry of Health, citizens can and do expect coherence: “[…] *almost two‐thirds of Danes point out that there is not enough coherence between the various parts of the healthcare system [and] Danes point to improved coherence as the most important issue for a better healthcare system*” (D1, p. 13). The healthcare system is constructed as fluid and seamless and bound together by digital information (D3, D4). Health professionals are constructed as responsible for carrying and providing this digital information through the EHR: “*It should not be up to the individual patient or their relatives to carry information about treatment and history throughout the healthcare system. Citizens–patients and relatives–must be able to expect the healthcare professionals they meet to know the relevant information*” (D1, p. 18). The citizen is constructed as a consumer who has the right to expect and demand certain health services delivered from the healthcare system. Good service or good quality is equivalent to a seamless system. Moreover, the health professionals' job is to ensure this seamless experience.

#### 4.2.2 The governed health professional

A second effect is that health professionals are constructed as governed by management. The importance of standardized documentation practice is bound by a managerial interest in valid data (Kommunernes Landsforening, 2010b), and is based on the conceptual logic that digitalization creates more value for money (Regeringen et al., 2007). The CLP document constructs an organizational hierarchy between health professionals, signifying that documentation practices are independent of professional competencies. The hierarchy is described by consistent use of two organizational roles: "*public authority*” and “*supplier*” (D2, p. 8). The authorities are described as being *“*[…] *responsible for follow‐up and assessment of the expected condition(s)”* (D2, p. 11). According to the documents, the public authority carries the organizational role of concrete daily prioritization within the given framework of the organization. In contrast, the supplier's task is to receive orders, and plan and execute the services. The supplier is not given responsibility for assessment or prioritization, but “[…] *must ensure that the citizen's conditions and instructions for action are updated if there are changes in the citizen's situation. Follow‐up by the authority depends on the citizen's information being updated*” (D2, p. 12).

Thus, the health professional is placed in a subject position as an actor that represents an organization, and not representing a specific profession. Furthermore, with the CLP, documentation practice is framed as an organizational activity in contrast to documentation as a health professional activity defined by professional knowledge, norms, and values.

#### 4.2.3 Standardization

Central elements in standardization are “*health conditions*” and “*functional conditions*” (D2, p. 22), which are described in the CLP document but not in the digitalization strategy or inspection documents. The two categories define what is valuable and what can be assessed and documented because all documentation is structured according to a unique predefined structure of conditions. It is impossible to choose unspecified conditions if the citizens' need for treatment or care does not fit the classification. For example, a municipal care trajectory is defined by 43 health conditions and 30 functional conditions depending on which defined services the citizen is entitled to receive. In addition, a care trajectory has to be evaluated, and the expected end date for the service must be stated (D2). Strictly professional documentation, only for professionals' use, is described as “*documentation for the sake of documentation*” (Kommunernes Landsforening, 2002, p. 10), which implies that documentation without a managerial purpose is a waste of valuable time. A discursive effect is that previous professional documentation practice (nonstandardized and patient‐oriented) appears to be inappropriate. The “right kind” of documentation is constructed as short, strict, and reflecting the correct classifications. The classifications implemented with the introduction of digital systems contribute to a specific form of standardization of professional language. The term “*minimum data set*” (D2, p. 17) is used to characterize some kinds of documentation as being more significant than others. Observations or actions based on the health professional's tacit knowledge or professional gaze become nonexistent, as there is no room for this in the EHR.

Thus, the categories construct a “task‐oriented” practice, framing healthcare practice through certain activities produced by health conditions. It is only possible to assess citizens' needs through predefined conditions, and it is impossible to assign a task if it does not fit the predefined categories.

## 5 DISCUSSION

Problem representations implicate materializations “into the real” (Bacchi & Goodwin, 2016). In our case, several subtypes of standardization materialize in municipal healthcare. First, the CLP defines a uniform nationwide design of the EHR. Second, the CLP's specific terminology builds upon certain classifications (D2, p. 15). Third, the CLP performance standards define specific roles and tasks, creating procedural standardization, and specifying which actions are appropriate at particular times (D2). Fourth, the Quality Control documents act as performance standards, providing indicators for quality control (D3, D4). The overall effect is that the digital documentation functions as organizational micromanagement (Cleary et al., 2015) of municipal healthcare to create value (Lega et al., 2013). The health professionals are offered the subject position of "deliverer" or "employee,” and procedural standardization operates as an organizational tool to guide concrete workflows and healthcare services, leading to what other researchers have termed a LEAN‐line practice (Crema & Verbano, 2016; Hung et al., 2021). In general, health professionals are characterized as performing their duties while having dual loyalty to the state and citizen, while their practice is guided by a profession‐specific normative framework (Grimen, 2006; Hjort, 2012). According to Kijne and Frederiksen (2019), health professionals' normative framework centers on the concept of caring and is based on an implicit contract to behave according to certain norms resulting in unspoken expectations about actions (Andersen, 2009). As such, health professionals are stuck between professional ideas of documentation, and the governmental discourse of efficiency, standardization, and anonymous organizational roles.

The quality control documents construct the idea that health professionals bear sole responsibility for assessments, workflows, and professional language. In contrast, the method handbook constructs concrete practice guidelines in an organizational language that is oriented towards efficiency. Thus, health professionals seem to be caught between a rock and a hard place in an EHR‐system that offers only the role of organizational employee supplying standardized services and quality control based on professional autonomy and responsibility. The dilemma is consistent with former research in policy and discourse that reveals conflicts and paradoxes in municipal healthcare (Dahl, 2019; Østensen et al., 2019). The professionals' voice is absent from the practical documents, which may lead to the assumption that the professionals take a subordinate position, allowing governmental problem representations to achieve legitimacy. As such, it seems that the subject of digital documentation is framed by governmental actors, deploying discourse on documentation for managerial purposes. Even though the selected documents in this analysis could indicate governmental control of digital documentation in healthcare, it is important to remember that “we are all subjects constituted in discourse” (Bacchi, 2009, p. 237), and this creates space for challenging that discourse in practice. Wilhelm (2020) points out that standardizing work in healthcare takes place in highly complex settings and can be both actively resisted or actively maintained, depending on the type of work and social situation. In addition, Taggart (2015) discusses difficulties with achieving structured data in EHRs due to "staff inertia." This could indicate that discourses regarding digital documentation, as well as its effects, are still up for negotiation.

Research indicates that standardization of services and procedures improves patient safety and efficiency. For example, standardization of clinical pathways can reduce harm and death (Borovac‐Pinheiro et al., 2021; Schmitz et al., 2021; Turner et al., 2021), refine clinical outcomes and quality of treatment (de Belvis et al., 2019), and improve efficiency (Joris et al., 2015) and organizational outcomes, for example, length of stay (Lagergren et al., 2020; Leonard et al., 2016). Nonetheless, the standardization of services with extensive use of categories in the CLP risks a fragmented system, focusing on the individual task (by the minute) with the risk that the overview and coherence of the citizen disappear. Many citizens have longitudinal trajectories in municipal healthcare across legislations, organizational units, and employees with different professional backgrounds (Bødker; & Glasdam, 2021; Møller & Delmar, 2019; Rasmussen; et al., 2021). The combined effect of a narrow focus on tasks and anonymous health professionals is that the singular care meeting is at risk of becoming an impersonal meeting between consumer and provider.

### 5.1 Implications for further research

This study provides insights into how problem representations operate at microlevel in the form of discursive subjectification, and lived effects. It indicates that municipal healthcare practice transforms due to these effects. We propose that it is time for critical reflection on the effects of standardization. This requires further research into how digital documentation changes organizational priorities and work processes, the professionalism of the health profession, and coherence for citizens: for example, how health professionals interact with citizens whose needs do not fit into predefined categories, or how professional autonomy is maintained in a system offering only the role of the organizational employee who supplies standardized services.

### 5.2 Strengths and limitations

In this study, the object of investigation was documents. In accordance with the WPR approach, we have suggested possible effects of municipal care practice resulting from this way of presenting digital documentation. However, the approach cannot account for the concrete effects of implementing digital documentation.

The WPR approach is open to various analytical strategies. For instance, Arousell (2017) applied all questions to identify logic in an interview study, Walker (2020) emphasized two of the questions in an interview analysis, and Skovhus (2017) identified problem categories before analyzing documents. Our study included all questions to analyze the topic in multiple dimensions, including genealogical features, rationalities, and potential effects. As such, we have performed an integrated analysis by applying all questions to uncover a “tightly woven” representation of problem representations. Using poststructuralism as the theoretical basis for analysis, it becomes clear how practical documents not only represent the subject of digital documentation but also take part in producing digital documentation practices, bridging from text to possible real effects.

## 6 CONCLUSION

Our study revealed three problem representations: lack of coherence in a complex healthcare system, lack of assessable data for management and political institutions to prioritize health services, and an inefficient healthcare system. Digital healthcare documentation stands uncontested as the solution to all three problem representations. Digital documentation co‐constructs a fully standardized healthcare practice, covering a uniform design, specific terminology, well‐described processes, and quality control by performance standards. Healthcare documentation is considered an essential part of healthcare practice; however, the standardized categories and classifications limit the language, practice, and detailed individualized healthcare. Thus, digital documentation can be understood as an organizational micromanagement approach as it assigns the health professional a subject position as an employee acting according to the organization's framework, instead of the profession's normative framework. Municipal healthcare is at risk of being fragmented due to digital documentation's focus on the organizational management of health with a task‐oriented practice supplied by an anonymous health professional.

## CONFLICT OF INTEREST

The authors declare no conflicts of interest.

## Supporting information

Supporting information.

## Data Availability

Data sharing not applicable to this article as no datasets were generated or analyzed during the current study.

## Footnotes

1 Original quotations translated by the authors.
