# Supplementary material for: How digital health documentation transforms professional practices in primary healthcare in Denmark: A WPR document analysis
Source: Nurs Inq. 2022 May 10;30(1):e12499. doi: 10.1111/nin.12499 (PMC10078429; doi:10.1111/nin.12499)
Supplement: Supplementary file 1 — Supporting information. [file NIN-30-0-s001.docx]

Supplementary information ^[[1]](#footnote-1)^

| Published | Title | Author |
| --- | --- | --- |
| 1999 | National Strategi for IT i Sygehusvæsenet 2000-2002  [National Strategy for IT in the Hospital System 2000-2002] | The Health Ministry |
| 2002 | På vej mod digital forvaltning – vision og strategi for den offentlige sektor  [Towards digital governance – vision and strategy for the public sector] | Digital Management  Government  KL- LGD^[[2]](#footnote-2)^  County Council  Municipality of Copenhagen  Municipality of Frederiksberg |
| 2002 | Værd at vide om fælles sprog  [Worth knowing about Common Language] | KL – LGD |
| 2004 | Strategi for digital forvaltning 2001-2006  [Digital management strategy 2001-2006] | Government  KL- LGD  Digital Government  County Council  Municipality of Copenhagen  Municipality of Frederiksberg |
| 2004 | Fællessprog II  [Common Language II] | KL – LGD |
| 2007 | Strategi for digitalisering af den offentlige sektor – Mod bedre digital service, øget effektivisering og stærkere samarbejde  [Strategy for digitalization of the public sector – towards better digital service, increased efficiency and stronger collaboration] | Government  KL- LGD  Regional Denmark |
| 2008 | National Strategi for digitalisering af sundhedsvæsenet 2008-2012- til fremme af befolkningens sundhed samt forebyggelse og behandling  [National Strategy for digitalization of the Healthcare System 2008-2012 – To promote the population´s health, prevention and treatment] | Coherent digital Health in Denmark  Government  Regional Denmark  KL- LGD |
| 2008 | Registrering og dokumentation i hjemmeplejen – indtryk fra 7 kommuner  [Registration and documentation in home care - impressions from 7 municipalities] | FOA [Trade union]  Bureau 2000 |
| 2010 | Dokumentation og styring af hjemmesygepleje - Baggrundsnotat  [Documentation and management of home nursing- Background note] | KL- LGD |
| 2010 | Den Fælleskommunale Digitaliseringsstrategi 2010-2015 [Kort version]  [The Joint Municipal Digitization Strategy 2010-2015 [Short version] | KL- LGD |
| 2011 | Effektiv og Innovativ digitalisering af den offentlige sektor 2011-2015 – Handlingsplan for den fælleskommunale digitaliseringsstrategi 2011-2015  [Efficient and Innovative Digitalization of the Public Sector 2011-2015 - Action Plan for the Joint Municipal Digitalization Strategy 2011-2015] | KL- LGD |
| 2012 | Dokumentation af sygepleje – en statusrapport  [Documentation of nursing - a status report] | DASYS [Danish Nursing Association] |
| 2012 | Notat: Hvordan sikres kvaliteten i sygeplejen?  [Note: How is the quality of nursing ensured?] | KL- LGD |
| 2013 | Fællesoffentlig strategi for digital velfærd 2013-2020  Digital velfærd – en lettere hverdag  [Joint public strategy for digital welfare 2013-2020  Digital welfare - an easier everyday life] | Government  KL – LGD  Regional Denmark |
| 2013 | Notat: Ledelseshåndbog i god dokumentationspraksis  [Note: Management handbook in good documentation practice] | KL- LGD |
| 2013 | Notat: Vejledning til fælles konteringspraksis vedr. opgaver efter Sundhedslovens §138  [Note: Guide to common accounting practice regarding. tasks according to the Health Act §138] | KL- LGD |
| 2013 | Digitalisering med effekt – National strategi for digitalisering af sundhedsvæsenet 2013-2017  [Digitalization with effect - National strategy for digitalization of the healthcare system 2013-2017] | Government  KL- LGD  Regional Denmark |
| 2013 | Organisering af hjemmesygeplejen  [Organization of home nursing] | KL- LGD |
| 2013 | Notat: Afsluttende notat i Partnerskabsprojektet om hjemmesygepleje  [Note: Final note in the Partnership project on home nursing] | KL- LGD |
| 2013 | Vejledning om sygeplejefaglige optegnelser - VEJ nr 9019 af 15/01/2013  [Guidance on nursing professional records - INSTR no. 9019 of 15/01/2013] | The Health Ministry |
| 2014 | Notat: Delegation  [Note: Delegation] | KL- LGD |
| 2014 | Styring af hjemmesygeplejen – Inspirationsnotat  [Management of home nursing - Inspiration note] | KL- LGD |
| 2014 | Foranalyse: Muligheder for bedre brug af sundhedsdata  Transformationsplan  [Pre-analysis: Opportunities for better use of health data  Transformation plan] | Deloitte |
| 2015 | Vision: Sundhedsdataprogrammet  [Vision for the Health Data Program] | The Danish Health Data Authority |
| 2015 | Sundhedsprogrammets aktstøtte  [Health program act support] | The Health Ministry |
| 2016 | Fælleskommunal digitaliseringsstrategi 2016-2020  Lokal og digital- et sammenhængende Danmark | KL- LGD |
| 2016 | Digital understøttelse af tværgående komplekse patientforløb – sammenfattende rapport 2016  [Digital support of cross-disciplinary complex patient processes - summary report 2016] | The Danish Health Data Authority |
| 2017 | Sygeplejerskers dokumentationspraksis  [Nurses' documentation practice] | DASYS [Danish Nursing Association]  DSR [DNO-Danish Nursing Organization] |
| 2017 | Kommissorium – National bestyrelse for data på sundheds- og ældreområdet  [Terms of Reference - National Board for data in the field of health and the elderly] | Regional Denmark  KL- LGD  Ministry of children and elderly citizens  Ministry of Finance  The Health Ministry |
| 2018 | Rapport: Kulegravninger af ældrepleje og dagtilbudsområdet  [Report: Investigation of elderly care and the daycare area] | Agency for Digitalization  Rambøll |
| 2018 | Bekendtgørelse om autoriserede sundhedspersoners patientjournaler [journalføring, opbevaring, videregivelse og overdragelse m.v.]- BEK nr 530 af 24/05/2018  [Consolidation act on authorized healthcare professionals' patient records [record keeping, storage, disclosure, and transfer, etc.] – ACT no. 530 of 24/05/2018] | The Health Ministry |
| 2018  **D1** | Ét sikkert og sammenhængende sundhedsnetværk for alle – Strategi for digital sundhed 2018-2022  [One secure and coherent health network for all - Strategy for digital health 2018-2022] | The Health Ministry  The Ministry of Finance  KL- LGD  Regional Denmark |
| 2018  **D2** | Fællessprog III Metodehåndbog [vers. 1.61]  [Common Language Platform [vers. 1.61] | KL- LGD |
| 2018 | FSIII – standardisering af dokumentation i den kommunale hjemmepleje og hjemmesygepleje  [CLP - standardization of documentation in the municipal home care and home nursing] | KL- LGD |
| 2019 | Sundhedsdataprogrammet- Områdeanalyser  Kortlægning af kommunernes anvendelse af sundheds- og ældredata  [Health Data Program- Area Analyses  Mapping of the municipalities' use of health and elderly data] | KL- LGD |
| 2019  **D3** | Målepunkter for tilsyn på plejecentre, hjemmepleje, hjemmesygepleje, sygeplejeklinikker, akutfunktioner og midlertidige pladser 2019-2020  [Indicators for inspection at care centers, home care, home nursing, nursing clinics, emergency functions, and temporary places 2019-2020] | The Danish patient Safety Authority |
| 2019  **D4** | Målepunkter til Ældretilsynet på plejecentre, hjemmeplejeenheder og midlertidige pladser  [Indicators for the Danish Elderly inspection at care centers, home care units, and temporary places] | The Danish patient Safety Authority |
| 2020 | Notat: Projektbeskrivelse for synlige kommunale sundheds- og ældredata, version 4  [Note: Project description for a visible municipal health and elderly data, version 4] | KL-LGD |
| 2020 | Fremtidens digitale kompetencer – når arbejdslivet bliver mere og mere digitalt i kommunerne  [The digital competencies of the future - when working life becomes more and more digital in the municipalities] | KL- LGD |
| 2021 | Vejledning om sygeplejefaglig journalføring - VEJ nr 9521 af 01/07/2021  [Guidance on nursing professional records - INSTR no. 9521 of 01/07/2021] | The Health Ministry |

1. English translation by authors [↑](#footnote-ref-1)
2. KL- LGD: KL – Local Government Denmark [↑](#footnote-ref-2)
